# The Therapeutic Effects of Acorus Tatarinowii Volatile Oil and Electroacupuncture in Post‐Stroke Cognitive Impairment Patients: A Clinical Trial Protocol

**DOI:** 10.1002/brb3.70631

**Published:** 2025-07-03

**Authors:** Ying Dandan, Zhao Jingting, Wang Minzhe, Cai Zhuoyue, Li Xinyun

**Affiliations:** ^1^ Shaoxing Second Hospital Medical Community General Hospital Shaoxing Zhejiang China; ^2^ Center for Rehabilitation Medicine, Rehabilitation & Sports Medicine Research Institute of Zhejiang Province, Department of Rehabilitation Medicine, Zhejiang Provincial People's Hospital, Affiliated People's Hospital Hangzhou Medical College Hangzhou Zhejiang China; ^3^ School of Rehabilitation Hangzhou Medical College Hangzhou Zhejiang China

**Keywords:** cognitive, electric neck acupuncture, herbal olfactory therapy, RCT, stroke

## Abstract

**Background:**

Post‐stroke cognitive impairment (PSCI) is a common complication following stroke, with limited effective treatments. This randomized controlled trial aims to evaluate the efficacy of two non‐pharmacological interventions—electroacupuncture therapy (EA) and herbal olfactory therapy (HOT)—in improving cognitive function in PSCI patients, both as standalone treatments and in combination.

**Methods:**

This parallel‐group, assessor‐blinded, randomized controlled trial will recruit 210 PSCI patients, randomly assigned to one of six groups: control, EA, HOT, combination therapy, sham therapy, or the healthy (ratio 1:1:1:1:1:1). All participants will receive standard cognitive training and basic medical care. The EA group will receive 4 weeks of electro‐acupuncture at specific acupoints. The HOT group will receive 4 weeks of aromatic inhalation therapy using Acorus tatarinowii volatile oil. The combination group will receive both interventions. The primary outcome measure is cognitive function, assessed using the Montreal cognitive assessment (MoCA) at baseline, week 4, and week 8. Secondary outcomes include the mini‐mental state examination (MMSE), functional magnetic resonance imaging (fMRI), near‐infrared spectroscopy (fNIRS), and anxiety assessment using the state‐trait anxiety inventory (STAI) at baseline and week 4. Data analysis will be conducted using a modified intention‐to‐treat approach.

**Aims:**

This study aims to evaluate the clinical efficacy of EA at cervical acupoints combined with HOT for PSCI, with a specific focus on determining the therapeutic superiority of this combined approach over monotherapy interventions.

## Graphical abstract

Conceptual schematic illustrating the trial design and mechanisms. Electroacupuncture (EA) targets cervical acupoints (GB20/Gongxue) to improve cerebral blood flow and neural connectivity. HOT (herbal olfactory therapy) delivers Acorus Tatarinowii volatile oil via inhalation to penetrate the blood‐brain barrier, reduce neuroinflammation, and enhance synaptic plasticity. Combined therapy synergistically modulates LRPs/ARF1 signaling, promotes mitochondrial function, and clears Aβ plaques. Outcomes are assessed via MoCA/MMSE (cognition), fMRI/fNIRS (brain activity), and STAI (anxiety). Healthy controls provide normative brain function baselines.

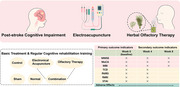

## 1 Introduction

In recent years, the global incidence, disability rate, and recurrence rate of stroke have remained high (GBD 2019 Stroke Collaborators 2021). Research data from various hospitals in China indicates that around 2 million people experience new‐onset strokes in the country annually (Wang et al. 2022). Stroke can result in neurological functional abnormalities, leading to cognitive, speech, physical, swallowing, and daily life functional impairments. Post‐stroke cognitive impairment (PSCI) is a clinical syndrome characterized by cognitive impairments persisting for up to 6 months after a stroke, with main clinical manifestations including varying degrees of impairment in attention, memory, orientation, perception, and executive function (Verdelho et al. 2021). The incidence of PSCI in stroke patients ranges from 17% to 92%, with 6% to 32% potentially progressing to dementia (Filler et al. 2024, Pendlebury and Rothwell 2009, Mahon et al. 2017). While medications for treating PSCI may offer some improvement in cognitive function, they also come with significant adverse effects (Shen et al. 2022). Consequently, the search for effective treatment strategies for post‐stroke cognitive impairment has become a key focus of research both domestically and internationally.

Traditional Chinese medicine treatment has been gaining attention, with aromatic opening drugs known for their strong, pleasant aroma and efficacy in treating conditions like central nervous system disease (Hou et al. 2021). Acorus Tatarinowii (ATR) contains various chemical components, including volatile oils, organic acids, terpenes, flavonoids, and amino acids. Despite volatile oils making up a small percentage (0.11‐0.42%) of ATR's composition, they are considered the key components for their pharmacological effects. Studies conducted in vitro and in vivo have highlighted the pharmacological activity of the active ingredients β‐asarone and α‐asarone in ATR's volatile oil, particularly in neurodegenerative diseases. These compounds have shown the ability to cross the blood‐brain barrier and exert rapid effects (Wang et al. 2023, Ning et al. 2024, Chellian et al. 2017).

Modern pharmacological studies have shown that ATR can protect central neurons, enhance memory and learning functions, resist nerve cell apoptosis, and inhibit central cholinergic activity, leading to significant therapeutic effects (Wang et al. 2023, Xu et al. 2023). Herbal olfactory therapy (HOT) using traditional Chinese medicinal materials triggers biochemical reactions in the respiratory, nervous, and circulatory systems, influencing human emotions, physiology, and behavior (Zhang et al. 2021). The volatile oil of ATR can quickly penetrate the blood‐brain barrier to provide therapeutic benefits. Moreover, certain acupuncture points have been found to stimulate brain activity and cognitive function. For example, Suliao (GV25) is known for its ability to clear heat and improve mental clarity, while Yingxiang (LI20) can enhance energy flow and cognitive function. By applying essential oils to these specific acupoints, the absorption of the oils can be enhanced, leading to improved therapeutic outcomes.

Electroacupuncture therapy (EA) involves connecting an electroacupuncture device to acupoints in the neck area and has been used in the clinical treatment of cerebral ischemia with significant therapeutic effects. Professor Weibin Gao proposed a novel treatment method for cerebrovascular diseases, which includes needling the bilateral Fengchi (GB20) and Gongxue to stimulate them with pulsed electric currents (Liu et al. 2017). This stimulation improves inadequate cerebral blood supply and enhances cerebral blood circulation. Electroacupuncture connecting the Fengchi (GB20) and Gongxue has shown noticeable effects in promoting meridian function, regulating metabolism, and improving limb and language functions in patients with head and neck diseases such as brainstem ischemia (Fu et al. 2023, Wang et al. 2009). This study aims to evaluate the clinical efficacy of electroacupuncture combined with ATR volatile oil inhalation therapy in treating PSCI. The research seeks to provide evidence that the integration of EA with ATR volatile oil inhalation therapy offers superior clinical benefits compared to single‐modality treatments for PSCI, through rigorous clinical investigation.

## 2 Methods

### 2.1 Objectives and Sample Size

**FIGURE 1:**
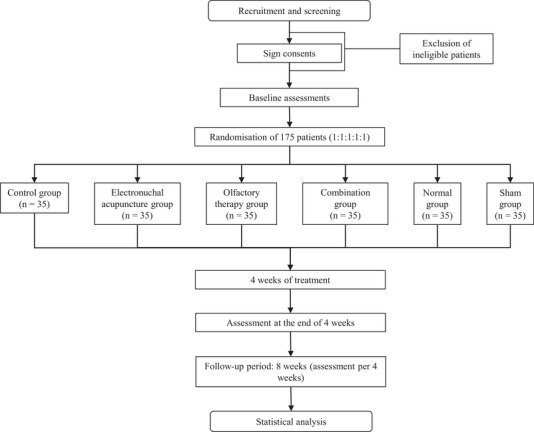
Flow diagram of the study.

This randomized controlled trial with 6 groups of therapists, assessors, and participants will be conducted from April 2024 to April 2025 in the Department of Rehabilitation Medicine at the Affiliated People's Hospital of Hangzhou Medical College. The study involves a 4‐week intervention for the enrolled patients. The study protocol was approved by the Institutional Review Board of the Affiliated People's Hospital of Hangzhou Medical College and was registered at https://register/clinicaltrials.gov(NCT06313866). Voluntary written informed consent will be obtained from all participants before entering the trial. We reported the trial according to the Consolidated Standards of Reporting Trials (CONSORT) statement (Figure 1).

The sample size for this study will be calculated using the formula for a superiority trial of two independent sample sizes. Clinical experience and preliminary research indicated an effective rate of 90% in the treatment group and 80% in the control group (Hei et al. 2023, Su et al. 2023, Zhang et al. 2023). Based on these data, we conducted a sample size calculation using a two‐tailed *t*‐test with the following parameters: test boundary value *δ* = 0.15, significance level *α* = 0.025, and power *β* = 0.20 (Zabor et al. 2020). The calculation was performed using the following formula.

$$n=\left[{\left(Z_{\alpha /2}+Z\beta \right)}^{\wedge 2}*\left(p1\left(1-p1\right)+p2\left(1-p2\right)\right)\right]/{\left(\delta -|p1-p2|\right)}^{\wedge 2}$$

Considering a case number ratio of k = 1 between groups, the calculated sample size was 32 cases per group. To account for a potential 10% dropout rate, we increased the sample size to approximately 35 cases per group (International Council for Harmonisation of Technical Requirements for Pharmaceuticals for Human Use 1998). The total sample will be divided into 6 groups: control, EA, HOT, combination therapy, sham therapy, or the healthy, totaling around 210 cases to be included.

### 2.2 Inclusion Criteria

1. Stroke as per the Traditional Chinese Medicine (TCM) definition, presenting symptoms include unilateral paresis or paralysis, sensory deficits, speech impairment, and hemianopsia. PSCI diagnosis involves clinically significant deficits in at least one cognitive domain and severe disruption of instrumental ADLs/ADLs.
2. The scores on the MMSE scale must adhere to the following criteria: Less than 17 points for individuals with illiteracy, less than 20 points for those with primary school education, and less than 24 points for individuals with a middle school education or higher.
3. The age range is 45 to 70 years;
4. No history of mental illness, clear consciousness, stable vital signs, and able to complete the scale assessment;
5. Within 3–6 months post‐stroke, diagnosed as a patient in the recovery period of cerebral infarction (or cerebral hemorrhage) at admission;
6. Signed informed consent by the patient or their family.

### 2.3 Excluded Criteria

1. Transient ischemic attack;
2. Subarachnoid hemorrhage;
3. History of severe liver or kidney diseases, mental illness, epilepsy, asthma, or obstructive pulmonary diseases;
4. Occurrence of cognitive impairment prior to stroke;
5. Severe communication barriers;
6. Substance abuse or heavy alcohol consumption;
7. Implanted cardiac pacemakers or other electronic devices.

### 2.4 Randomization and Blinding

Using a random number table method, the PSCI participants in the trial will be randomly allocated to (1) the control group, (2) the EA group, (3) the HOT group, (4) the combination group, (5) the sham group, and (6) the healthy group, with 35 individuals in each group. Notably, the present study was designed as a separate cohort of healthy subjects to assess brain functional abnormalities in patients with PSCI using functional magnetic resonance imaging (fMRI) and to validate the effects of interventions such as acupuncture and ATR volatile oil therapy on brain function. Data from healthy people provide a baseline of normal brain function for the study, helping to identify pathological changes in stroke patients. The allocation process is concealed, and the assessors will be kept blinded to the participant's allocation. Participants are instructed not to discuss their research participation or experiences with the assessors during the evaluation period. Patients will be informed that they would receive either EA treatment, HOT, or both, without being informed about the number of acupoints or the specific stimulation sites.

Acupuncture and herbal treatments will be administered by licensed acupuncturists, while a qualified rehabilitation physician will train a research assessor to evaluate the efficacy of acupuncture. The research assessor and data analysts will remain blinded to group assignment, although participants will be informed during the informed consent process.

### 2.5 Allocation Concealment

#### 2.5.1 Generation of Random Allocation Sequence

1. Use of randomization tools: A random number table or equivalent randomization tool was employed to generate a numerical randomization sequence.
2. Group assignment: Each random number was uniquely mapped to a participant ID and subsequently designated as either the experimental group or control group. This process was exclusively conducted and documented by authorized personnel.

#### 2.5.2 Preparation and Preservation of Random Allocation Table

1. Table creation: A formal “Random Allocation Table” was created to systematically record grouping information. The table was prepared in triplicate to ensure data integrity and redundancy.
2. Secure storage: The random allocation table was strictly preserved using multiple security measures, including encrypted digital storage and physical safeguards (e.g., locked cabinets), to prevent unauthorized access.

#### 2.5.3 Implementation of Allocation Concealment

1. Opaque sealed envelopes/containers: The allocation information was sealed in sequentially numbered, opaque envelopes to prevent premature disclosure of group assignments.
2. Custodian protocol: Three key stakeholders the principal investigator, acupuncture therapists, and statisticians—were each entrusted with separate copies of the allocation table. Simultaneous authorization from all custodians was required for any disclosure.

#### 2.5.4 Unblinding and Verification Procedures

1. Unblinding protocol: Group allocation information was only revealed through the synchronized opening of all sealed copies of the allocation table, ensuring temporal alignment and procedural transparency.
2. Allocation validation: Actual group assignments were systematically cross‐verified against the original randomization records through independent auditing, confirming compliance with the predetermined allocation scheme.

### 2.6 Intervention

#### 2.6.1 Control Group

Given that a considerable proportion of patients will require targeted treatment for underlying conditions such as hypertension, glycemic control, anticoagulation, and lipid regulation, concomitant therapies will be permitted during the study period. Moreover, each patient also underwent cognitive rehabilitation training five days a week with two days of rest for a total of 4 weeks. The standard drug treatment group was the control group. The enrolled patients will receive 5 mg of donepezil hydrochloride per night initially, which is increased to 10 mg per night after 30 days in the absence of any adverse reactions. The total treatment duration is 4 weeks.

#### 2.6.2 Electroacupuncture Therapy

**FIGURE 2:**
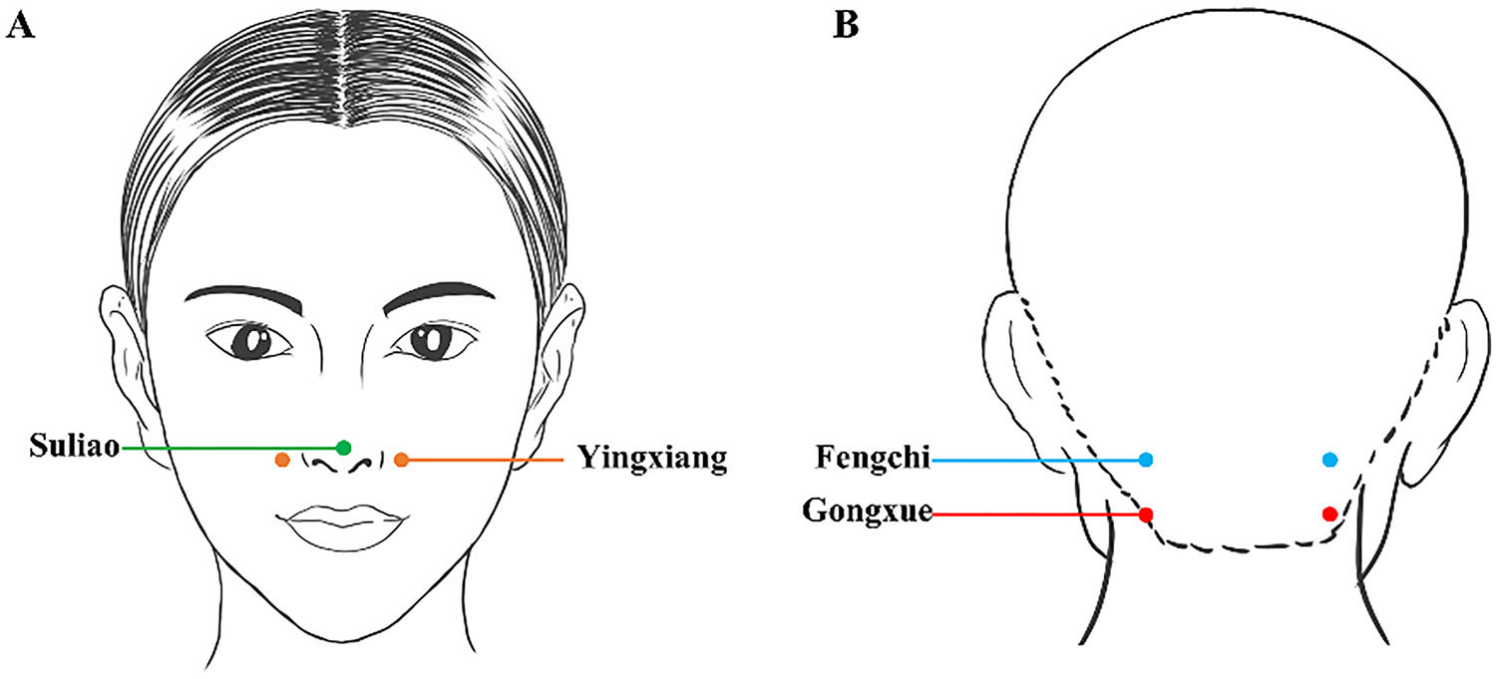
The acupoints used in olfactory therapy and electronuchal acupuncture. **(A)** The points displayed on the face, green: Suliao (GV25); orange: Yingxiang (LI20); and **(B)** Acupoints shown behind the head, blue: Fengchi (GB20); red: Gongxue.

All participants who will be recruited for the study will have no previous exposure to psychotropic medication treatment. Treatment involved using sterile 0.25×50 mm stainless steel Hua Tuo brand filiform needles and the KWD‐808II universal pulse electrotherapy apparatus while patients were seated. The acupuncturist will needle the bilateral Fengchi (GB20) and Gongxue, located 1.5 cm below Fengchi (GB20) (General nomenclature of science of acupuncture and moxibustion 2013). Hands and the needling area will be disinfected with alcohol before needling. Needles will be inserted into the acupoints with a slight downward and toward the throat direction, reaching a depth of 20–25 mm. Electrotherapy will be applied to the Fengchi (GB20) and Gongxue (Figure 2B, Table 1) on the same side of the head, with sparse waves causing slight head movement (Liu et al. 2017). The current intensity will be adjusted to patient tolerance for 30 min per session. This procedure will be done once daily for four courses of treatment lasting one week each. To maintain treatment consistency, the same therapist will perform all acupuncture interventions for the patients.

**TABLE 1:** Acupoints selected for use in the study.

Acupoints	Location	Manipulation
Fengchi (GB20) (bilateral)	On the nape, below the occipital, on a level with Fengfu (DU‐16), in the depression between the upper portion of the trapezius and the sternocleidomastoid	For normal manipulation, electric acupuncture will be used between the left Fengchi and Gongxue and the right Fengchi and Gongxue.
Gongxue (bilateral)	On the nape, located 1.5 inches straight down from Fengchi, level with the lower lip	For normal manipulation, electric acupuncture will be used between the left Fengchi and Gongxue and the right Fengchi and Gongxue.
Suliao (GV25)	On the human face, in the center of the tip of the nose.	Essential oil from ATR in a rollerball inhaler was applied uniformly to Suliao.
Yingxiang (LI20) (bilateral)	Open at the midpoint of the outer edge of the nose when it is in the nasolabial fold.	Essential oil from ATR in a rollerball inhaler applied uniformly to Yingxiang.

#### 2.6.3 Herbal Olfactory Therapy

The baseline characteristics of the enrolled patients will be similar to those described in Section 2.5.2 (as above). Following enrollment, participants will receive an immediate traditional Chinese medicine olfactory diagnosis treatment conducted by a single therapist to eliminate potential biases. The therapy will involve using essential oil from ATR in a rollerball inhaler, applied uniformly to specific acupoints (Figure 2A, Table 1, Suliao (GV25), bilateral Yingxiang (LI20) (Zabor et al. 2020)) to enhance cognitive awakening. Participants will be instructed to inhale the oil for 5 to 10 min, three to five times daily, for 4 weeks while maintaining steady and regulated breathing.

#### 2.6.4 Combination Group

The combination group will receive electroneutral acupuncture and olfactory therapy. The procedures were the same as in Sections 2.6.2 and 2.6.3.

#### 2.6.5 The Healthy Group

Healthy participants will receive an immediate traditional Chinese medicine olfactory diagnosis treatment conducted by a single therapist to eliminate potential biases. The procedure is the same as in Section 2.6.1.

#### 2.6.6 Sham Group

In the sham stimulation group, non‐penetrating acupuncture will make the patients experience a feeling similar to acupuncture. But it doesn't pierce the skin. Other treatments will be the same as Section 2.6.5.

### 2.7 Clinical Assessment

#### 2.7.1 Primary Outcomes

Participants' cognitive abilities will be assessed at three‐time points: baseline (week 0), during treatment (week 4), and follow‐up (week 8) (Table 2). The primary outcome measures include the Montreal Cognitive Assessment (MoCA), which are commonly used tools for evaluating cognitive function across different types of impairment.

**TABLE 2:** Schedule of enrollment, intervention, and assessment.

	Week 0 (baseline)	Week 4	Week 8
MMSE	X	X	X
MoCA	X	X	X
MBI	X	X	X
TCD	X	X	
fNIRS	X	X	
fMRI	X	X	
STAI	X	X	
Adverse Effects	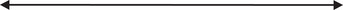		

#### 2.7.2 Secondary Outcomes

1. The mini‐mental state examination (MMSE): MMSE score will be assessed at three‐time points: baseline (week 0), during treatment (week 4), and follow‐up (week 8).
2. The Modified Barthel Index (MBI): MBI scores will be utilized to evaluate patients' activities of daily living and their level of dependency. It will be assessed at three‐time points: baseline (week 0), during treatment (week 4), and follow‐up (week 8).
3. Transcranial Doppler (TCD)

TCD involves the following steps: First, prepare the equipment, including the transcranial Doppler ultrasound device and the probe, ensuring that the device is properly calibrated and the appropriate parameters are set. Second, prepare the patient or subject, who is typically placed in a supine or sitting position, with ultrasound coupling gel applied to the scalp to ensure sound wave conduction, and the detection points are marked. Finally, detect the cerebral vessels by selecting the appropriate detection window (such as the temporal window, occipital window, and ophthalmic artery window), and adjust the probe angle and position to locate the target cerebral vessels.

Parameter settings: The light source wavelength typically employed ranges from near‐infrared light of 650 to 900 nm, as this range exhibits low absorption of light by both hemoglobin and water. The light source can be either a pulsed laser or an ultra‐short pulsed laser. Detection is performed using a Time‐Correlated Single Photon Counting (TCSPC) detector, which allows for time resolution at the picosecond (ps) level to measure the time of flight of photons. The probe layout generally maintains a distance of 2 to 4 cm between the light source and the detector.

Analysis method: Temporal point spread function (TPSF): This method involves analyzing the time‐of‐flight distribution of photons to derive the optical characteristics of tissue, specifically the absorption coefficient and scattering coefficient. Differential Pathlength Factor (DPF): This factor is employed to correct the path length of photons traveling through tissue. Calculation of Hemoglobin Concentration: The concentration changes of oxygenated hemoglobin (HbO2) and deoxygenated hemoglobin (HbR) are calculated based on variations in the absorption spectrum.

4 Near‐infrared spectroscopy (fNIRS)

fNIRS is a non‐invasive neuroimaging technique that evaluates regional blood oxygen metabolism in the brain by emitting near‐infrared light and detecting its scattered signals within brain tissue. When using fNIRS, the first step is to prepare the equipment, including near‐infrared light sources and detectors, and ensure proper device calibration. Participants are typically positioned in a comfortable seated or supine position, with their scalp cleaned to minimize light wave interference. The light sources and detectors are then secured at specific brain regions (e.g., the frontal or parietal lobes) using headbands or fixation devices to maintain stability. During data acquisition, the device records changes in the concentrations of oxygenated hemoglobin (oxy‐Hb) and deoxygenated hemoglobin (deoxy‐Hb) in brain tissue, reflecting hemodynamic dynamics associated with brain activity.

Light source wavelength: Typically, two or more wavelengths, such as 750 nm and 850 nm, are employed to differentiate between oxyhemoglobin (HbO2) and deoxyhemoglobin (HbR). Light source type: Continuous wave lasers or LEDs are commonly utilized. Sampling rate: A frequency range of 10–50 Hz is generally adopted to effectively capture the dynamic changes in blood oxygen levels. Probe layout: The distance between the light source and the detector is maintained at 2–3 cm to ensure accurate detection of cerebral cortex activity. Number of channels: Depending on experimental requirements, the system may incorporate 16 channels, 32 channels, or more.

The analysis method involves a revised application of Beer‐Lambert's law, which is utilized to convert variations in light intensity into corresponding changes in hemoglobin concentration. Baseline correction is employed to eliminate low‐frequency drift and high‐frequency noise from the signal. Task‐related signal extraction involves the extraction of blood oxygen signals associated with specific tasks, utilizing either the general linear model (GLM) or an event‐related averaging method, akin to ERP analysis. Finally, functional connectivity analysis is conducted to calculate the functional connections between different brain regions, employing metrics such as the Pearson correlation coefficient or phase synchronization.

5 Functional magnetic resonance imaging (fMRI)

fMRI is a high spatial resolution neuroimaging technique that maps brain functional activity by detecting blood‐oxygen level‐dependent (BOLD) signals.

Parameter settings for the imaging process include a magnetic field strength typically set at 3 Tesla (T), although devices with field strengths of 7T or higher are also available. The scan sequence utilizes a gradient echo planar imaging (EPI) sequence to capture blood‐oxygen‐level‐dependent (BOLD) signals. The time resolution (TR) is 3 s. The spatial resolution is typically 2 mm isotropic voxel. Scanning is conducted in a manner that encompasses the entire cerebral cortex through whole‐brain scans.

The analysis method involves several preprocessing steps: Realignment to remove head motion artifacts, normalization to map the individual brain to a standard space (such as MNI space), and smoothing using a Gaussian kernel to enhance the signal‐to‐noise ratio. For resting state analysis, functional connectivity is assessed by calculating time series correlations between different brain regions. Additionally, Independent Component Analysis (ICA) is employed to extract resting state networks, such as the default mode network.

6 The state‐trait anxiety inventory (STAI)

State‐Trait Anxiety Inventory is a self‐report assessment of state anxiety and trait anxiety, including two subscales, State‐Trait Anxiety Inventory‐State (STAI‐S) and State‐Trait Anxiety Inventory‐Trait (STAI‐T). STAI‐S and STAI‐T are used for assessment the temporary condition of state anxiety and the more general and long‐standing quality of trait anxiety, respectively. Each subscale consists of 20 items. The STAI‐S scale is rated on 4‐point likert scale (1 = not at all, 2 = somewhat, 3 = moderately and 4 = very much). Each STAI‐T item is rated on a 4‐point Likert scale as well (1 = rarely, 2 = sometimes, 3 = often and 4 = almost always). STAI has been shown to have good internal consistency and test‐retest reliability.

### 2.8 Data Security and Monitoring

The data management of this study is overseen by the research team to uphold the authenticity, integrity, confidentiality, and traceability of clinical trial data. The Case Report Form (CRF) will be completed by the principal investigator or other authorized researchers, with only qualified medical researchers permitted to input raw clinical assessment/safety data. Any modifications made to the raw data on the CRF by the principal investigator or other authorized researchers will be recorded. Approved data modifications necessitate the researcher making the changes by signing their name, indicating the modification date, and providing the rationale for the modification. Researchers will adhere to standard operating procedures to ensure quality control of the clinical trial and the implementation of a quality assurance system. All observed results and findings in the clinical trial will be validated to guarantee the data's reliability and to ensure that all conclusions drawn from the clinical trial are based on original data. Quality control measures will be enforced throughout the data processing stages to verify the reliability and accuracy of all processed data.

### 2.9 Safety Monitoring

Acupuncture and olfactory therapy pose mild adverse effects. Acupuncture may cause pain, numbness, or bruising, while olfactory therapy may lead to allergic reactions or affect the liver or kidneys. Serious adverse events will be promptly reported, and patients experiencing such events will be withdrawn from the study. Adverse events will be documented on case report forms and reported to statisticians.

Adverse events (AEs) are typically classified into three severity grades: mild, moderate, and severe. Mild adverse events refer to symptoms that are tolerable to participants, requiring no specific intervention, and do not affect the trial progression or participant prognosis. Moderate adverse events involve clinically significant symptoms that are difficult for participants to tolerate, potentially necessitating trial discontinuation or medical treatment, and may impact disease recovery. Severe adverse events are life‐threatening conditions that could lead to permanent disability or mortality, requiring immediate trial termination and emergency medical intervention. During the treatment period, any adverse events must be documented in real‐time, investigated for causality, and promptly reported to the supervising physician for evaluation and management.

### 2.10 Quality Control

Experienced and licensed physicians and therapists will administer interventions after receiving regular two‐day training sessions on treatment options. Standard operating procedures (SOPs) will be developed for participant screening, randomization, blinding, and manipulation to ensure consistency among investigators.

### 2.11 Data Analysis

Researchers who are blind to the groupings will conduct data management and statistical analysis on the data acquired using the statistical software SPSS 22.0 (SPSS Inc., Chicago, IL, United States). Descriptive statistics will be utilized to depict the baseline characteristics of the two participant groups, with mean ± standard deviation for normally distributed continuous data and median and quartiles for non‐normally distributed continuous data. The normality of the data will be assessed using the Shapiro–Wilk test, and the homogeneity of variance will be assessed using the Levene test. One‐way analysis of variance (ANOVA) will be used for comparisons among multiple groups for normally distributed continuous data, while repeated measures analysis of variance will be used for within‐group pre‐ and post‐treatment comparisons. The Kruskal–Wallis test will be employed for non‐normally distributed continuous data. The significance level (α) for the statistical hypothesis tests will be set at *p* < 0.05, one‐tailed.

## 3 Discussion

PSCI is a form of cognitive dysfunction that can occur following a stroke. Two non‐pharmacological treatment methods that have gained attention for their minimal side effects are traditional Chinese medicine olfactory inhalation therapy and electric neck acupuncture. Electric acupuncture has been widely used in treating neurological conditions like cerebral ischemia, Alzheimer's disease, and Parkinson's disease (Cai et al. 2019, Shin et al. 2017, Kong et al. 2010). Electric neck acupuncture, a novel technique developed by Professor Gao Weibin, leverages the anatomical, physiological, and pathological features of the neck to treat neurological disorders effectively (Liu et al. 2017). While there is limited research on the efficacy of electric neck acupuncture for PSCI, TCM olfactory inhalation therapy focuses on enhancing cognitive function and emotional well‐being by stimulating the olfactory nerve in brain regions associated with the sense of smell. Ancient texts like the Divine Farmer's Materia Medica mention the therapeutic benefits of olfactory inhalation therapy for conditions such as senile dementia, stroke, and epilepsy. HOT works through the aroma molecules of plants, which are transmitted to the limbic system of the brain and even through the pores of the human body, penetrating into the deeper tissues, resulting in muscle relaxation and a pleasant emotional feeling. Studies have shown that the use of calamus volatile oil by gavage for three weeks can reduce nitric oxide synthase (NOS) in the brain and hippocampus of Alzheimer's disease mouse models, and significantly improve learning and memory dysfunction (Tian et al. 2012). Inhalation of eugenol over a 4‐week period enhanced learning memory by interfering with neurotransmitters in the olfactory system, thereby altering olfactory afferent signals (Shuai et al. 2021). Our study aims to explore the potential of traditional Chinese medicine olfactory inhalation therapy and electric neck acupuncture, either individually or in combination, as treatments for PSCI.

Our research aims to evaluate the improvement of cognitive function in individuals with PSCI using different treatment methods. We have selected MoCA as the primary outcome measure for this study. MMSE and MBI is used as secondary outcome measures. The MoCA is another rapid screening tool for cognitive impairment, particularly sensitive to mild to moderate impairments, covering a wider range of cognitive domains comprehensively (Pinto et al. 2019). It includes more extensive memory tests by increasing the number and difficulty of words, extending the delay in recall, and providing a more accurate reflection of participants’ memory status. However, it requires more time compared to the MMSE. The MMSE is the most widely used dementia screening scale globally, focusing on cognitive abilities including orientation, memory, attention, calculation, recall, and language. It is a simple tool that aids in initial screening and categorizes participants based on their education level, with higher scores indicating stronger cognitive abilities, classified as normal, mild cognitive impairment, and dementia (Creavin et al. 2016). The ultimate goal of improving cognitive function is to reintegrate patients into their families and society. The MBI assesses daily living abilities in areas such as grooming, bathing, feeding, toileting, dressing, bowel and bladder control, stair climbing, transferring, walking on level surfaces, and wheelchair use. It provides different levels of ratings and reflects participants’ capabilities in daily activities (Liu et al. 2020).

In addition to the primary outcome measures, we also included some imaging examinations as secondary measures. fMRI is used to assess cerebral hemodynamics and identify activated brain regions to compare different groups (Logothetis 2008). fNIRS is a non‐invasive, portable, and cost‐effective method that measures changes in oxygenated and deoxygenated hemoglobin levels in the cerebral cortex using near‐infrared light, reflecting neural activity (Irani et al. 2007). fNIRS allows for convenient self‐control and observation of the brain during treatment, providing detailed insights into stimulated brain areas by acupuncture and Traditional Chinese medicine olfactory. Ultrasound Doppler is another non‐invasive method to examine cerebrovascular diseases by detecting hemodynamic and physiological changes in major intracranial arteries (Wan et al. 2022). These secondary measures help us understand participants' cerebral vascular function changes during treatment and offer preliminary evidence for further exploration of underlying mechanisms. Moreover, by comparing with the healthy population, this study more clearly revealed the abnormal brain function of patients with cognitive impairment after stroke and the effect of intervention on their brain function. This design not only enhances the scientific validity of the study results, but also provides a more reliable basis for clinical application.

The study design was limited to a single‐center study, resulting in a relatively small sample size. To enhance the robustness of the study, future studies should include more diverse cases from multiple centers. Additionally, the lack of a sham stimulation group as a control group due to controversies surrounding the need for sham acupuncture and its implementation is a limitation of this trial design. Subsequent experiments will delve into detailed basic research both in vitro and in vivo to uncover the molecular mechanisms behind HOT and EA treatment for enhancing cognitive function in stroke patients. While non‐pharmacological treatments show promise in addressing PSCI injuries, further extensive research and clinical trials are essential to validate their efficacy and safety. Therefore, the significance of this study lies in providing stronger evidence for the clinical application of these treatments.

## Author Contributions

**Ying Dandan**: validation, methodology, investigation. **Zhao Jingting**: writing – original draft, visualization. **Wang Minzhe**: data curation. **Cai Zhuoyue**: data curation. **Li Xinyun**: writing – review and editing, resources, project administration, funding acquisition.

## Ethics Statement

The study protocol was approved by the Institutional Review Board of the Affiliated People's Hospital of Hangzhou Medical College (KT2024016) and was registered at https://register.clinicaltrials.gov (NCT06313866), and registered on March 21, 2024.

## Consent

Informed consent was obtained from all individual participants included in the study.

## Conflicts of Interest

The authors declare no conflicts of interest.

## Peer Review

The peer review history for this article is available at https://publons.com/publon/10.1002/brb3.70631.

## Data Availability

The data that support the findings of this study are available upon request from the corresponding author, upon reasonable request.
